# Reward of labor coordination and hunting success in wild chimpanzees

**DOI:** 10.1038/s42003-018-0142-3

**Published:** 2018-09-10

**Authors:** Liran Samuni, Anna Preis, Tobias Deschner, Catherine Crockford, Roman M. Wittig

**Affiliations:** 1 Max Planck Institute for Evolutionary Anthropology, Deutscher Platz 6, 04103 Leipzig, Germany; 2 Taï Chimpanzee Project, Centre Suisse de Recherches Scientifiques, BP 1303, Abidjan, 01 Côte d’Ivoire

## Abstract

Cooperative hunting and meat sharing are hypothesized as fundamental to human life history adaptations and biological success. Wild chimpanzees also hunt in groups, and despite the potential of inferring ancestral hominid adaptations, it remains unclear whether chimpanzee hunting is a cooperative act. Here we show support for cooperative acquisition in wild chimpanzees since hunters are more likely to receive meat than bystanders, independent of begging effort. Engagement in prey searches and higher hunt participation independently increase hunting success, suggesting that coordination may improve motivation in joint tasks. We also find higher levels of urinary oxytocin after hunts and prey searches compared with controls. We conclude that chimpanzee hunting is cooperative, likely facilitated by behavioral and neuroendocrine mechanisms of coordination and reward. If group hunting has shaped humans’ life history traits, perhaps similar pressures acted upon life history patterns in the last common ancestor of human and chimpanzee.

## Abstract (web summary)

Liran Samuni et al. examined meat sharing behavior and oxytocin in wild chimpanzees, elucidating the mechanism facilitating cooperation. They find that hunters are more likely to receive food independent of begging, suggesting that prosocial behaviors are rewarded in primates, likely facilitated by oxytocin.

## Introduction

The human foraging niche, particularly group hunting and meat sharing, is central in debates regarding the evolution of human sociality and life history traits^1–4^. Humans’ hunting behavior is learning-intensive as it involves highly complex foraging skills acquired over one’s lifetime and which increase with age, allowing hunters to achieve maximum energetic return rates^4,5^. Superior hunting skills are linked to increased reproductive success in hunters, who are predominantly males^6,7^. Meat is a highly valuable food source in human diets, both in terms of energy and nutrition^8^, and the sharing of meat provides important social and energetic benefits, as it buffers variation in hunting returns and shortfalls^6,9^. Thus hunting and meat sharing are considered fundamental to human life history adaptations like long life spans, prolonged juvenile dependence, and increased survival^4^. Moreover, the sharing of meat may facilitate future participation in similar tasks by rewarding participants for their labor^10^. Such is posited in the cooperative acquisition hypothesis^10^, suggesting that sharing mainly occurs among participating hunters as a reward for their labor and not as an artifact of increased begging effort by hunters, thus potentially buffering variation in hunting return rates across hunters.

Hunting and meat sharing are often cooperative activities in humans and are maintained by various processes, such as kin selection, reward of labor, increased reputation, or reciprocity^11^. Although cooperative hunting has been observed in taxa such as lion-fish^12^, carnivores^13,14^, cetaceans^15,16^, and chimpanzees^17^, the complexity of the division of labor and the exchange of meat in humans appears unique^11^. Nonetheless, the extent to which our hominid ancestors relied on cooperation in group hunting and meat eating and the underlying mechanisms facilitating such group activities are still unclear^18^. Thus a comparative approach to assess whether group hunting and meat sharing are cooperative endeavors in other species and the mechanisms involved will contribute to our understanding of cooperative hunting and theories of selection pressures involved in human evolution.

Chimpanzees, one of our closest living relatives, hunt in groups and exchange meat with both kin and non-kin^17,19–25^. The degree to which chimpanzee hunting behavior is a cooperative act, in the sense that joint participation increases success and that hunters benefit more than bystanders^26^, is hotly debated and varies across populations^17,19,27,28^. In the Taï Forest, Côte d’Ivoire, patterns of meat sharing may stabilize cooperation, as hunters that successfully participated during the hunt gain more access to meat than non-hunters^17,25,29^; however, begging effort was not considered. Conversely, in other populations hunting appears more opportunistic^17,28–30^. Ecological and demographic factors, such as forest structure, prey species, and number of hunters, are thought to account for population differences in hunting frequency and success^19,20,22–25,31^, as well as the degree of coordination exhibited during a hunt^17,19^.

Coordinated activity is a pivotal component of humans’ cooperative acts and is considered particularly important in tasks like hunting that require dynamic responses to partner’s movements^32^. A growing body of observational and experimental studies in humans suggests that coordinated actions function to prime the motivation and socio-cognition required to jointly pursue goals with partners^32,33^, such as synchronous marching before combat^32^. However, it remains unclear whether joint participation in coordinated activity facilitates motivation in joint tasks in non-human animals.

In Ngogo^27^ and Taï^34^ field-sites, prior to initiating hunts individuals occasionally participate in pre-hunt searches for monkey prey or hunt patrols. A hunt patrol is characterized as a highly coordinated activity, during which individuals travel cohesively, slowly, with frequent pauses and rarely forage or vocalize (see Methods). Not all monkey groups encountered during hunt patrols are hunted, and the salient features of hunt patrols can continue, at times for hours, until a hunt is initiated. Although hunting patrols precede approximately 50% of hunting events in both Taï^34^ and Ngogo^19^, the contribution of hunt patrols to hunting success has rarely been studied, despite the potential contribution to our understanding of benefits of hunting behavior. The coordinated behavior observed during chimpanzee hunt patrols may serve as a behavioral mechanism that signals hunt motivation and enhances subsequent hunting success.

On a physiological level, the oxytocinergic system might influence participation in joint group activities, such as hunting, as the system is suggested to facilitate cooperative behavior^35–37^. Oxytocin is highly conserved across mammalian taxa in both neuroanatomy and functionality^35,38^. The oxytocinergic system operates both peripherally, by regulating parturition and lactation, and centrally, by affecting social behavior and social perception^39^. Oxytocin is suggested to facilitate cooperation both in and outside of reproductive contexts through increased tolerance and coordination^35–37^, supported by positive feedback through the brain’s reward centers^35^, although see ref. ^40^ detailing other effects of oxytocin on emotions and behavior. In chimpanzees, the coordinated behavior observed during both border patrols and group hunting involves increased oxytocinergic system activity in comparison to social and non-social control contexts^41^, and oxytocin may facilitate essential participation and social support in situations that are otherwise risky. This raises the question of whether chimpanzee hunt patrol behavior involves oxytocinergic system reactivity, potentially facilitating joint hunt participation, which may be highly beneficial if enhancing hunting success.

In sum, evidence to date suggests that cooperation in hunting is not a uniquely derived human trait. Therefore, we examined whether mechanisms considered unique to human hunting also contribute to chimpanzee group hunting. We examine the reward of labor and behavioral and neuroendocrine involvement during searches for monkey prey and hunting in males and females of two groups of habituated chimpanzees (*Pan troglodytes verus*) of the Taï Forest, Côte d’Ivoire. We find that hunt participation is the strongest predictor in influencing meat sharing, since hunters are overall more successful beggars than non-hunters independent of motivation in acquiring meat. We also find that both the number of hunters and participation in hunt patrols increases hunting success. Furthermore, we find increased oxytocinergic system activity after hunt patrols that is similar to levels after hunting activity, suggesting that oxytocin is associated with chimpanzee group hunting. Taken together, we find evidence in chimpanzees of the Taï forest for cooperation in group hunting, considered a key building block of the unique human foraging niche.

## Results

### Hunting and meat sharing in Taï chimpanzees

We observed two groups of chimpanzees (East and South) in the Taï National Park, Côte d’Ivoire over two field seasons. In total, we observed 143 hunts on 118 different days of which 80 cases were successful (56%) during the study period. East group individuals were successful in 58/107 (54%) hunt attempts, while South group individuals were successful in 22/36 (61%) hunt attempts. Group hunts, involving two or more hunters, were the majority (86%) of all hunts, with success rates for single hunters being at 16%, as opposed to 61% for group hunts. The majority of hunters were males (85%), with an average of 3.08 ± 1.48 (mean ± SD) hunters and 2.58 ± 2.55 bystanders per hunt. All hunting events involved the pursuit of monkeys, with successful hunts including bay red colobus (*Procolobus badius badius*; *n* = 58), western black and white colobus (*Colobus polykomos*; *n* = 17), lesser spotted-nosed guenon (*Cercopithecus petaurista*; *n* = 2), and mona guenon (*Cercopithecus mona*; *n* = 3).

Successful hunting events often attract other group members to the hunting area, and the average number of bystanders per meat sharing event was 3.86 ± 3.3. Not including individuals that caught the prey (catchers), the majority of meat was accessed via sharing (246/251 cases of accessing meat; 98%) and only 5 cases of scrounging were observed, all by bystanders (non-hunters) who had begged but did not receive a share. In total, 7 hunters and 71 bystanders did not receive a share of the meat, while 3 out of the 7 hunters and 54 out of the 71 bystanders did not receive a share despite begging for meat. Catchers fed on the meat in 100% of cases.

### Reward of labor

For 19 out of the 80 cases of successful hunting events, we were not able to reliably document who hunted either because the focal individual arrived to the hunting area after the monkey was captured (*n* = 10 hunts) or because we could not track the hunt movements of every adult individual due to forest visibility or low number of observers (*n* = 9 hunts). For 53 (87%) out of the remaining successful hunting cases, we documented the identity of hunters and all adult individuals that begged and accessed meat via sharing.

**Fig. 1:**
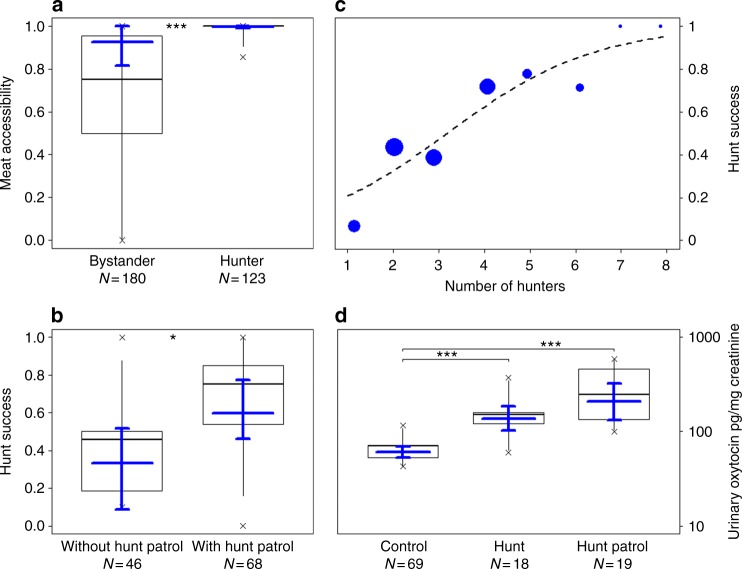
Effects of: **a** hunt participation on the likelihood to access meat in chimpanzees (331 data points; 32 subjects; 53 events), **b** hunt patrol behavior on hunt success in wild chimpanzees (112 events; 91 days), and **d** hunt patrol and hunting on urinary oxytocin levels in East group chimpanzees (106 samples; 10 subjects; 85 events). Shown are medians (thin horizontal lines), quartiles (boxes), percentiles (2.5 and 97.5%; vertical lines), minimum and maximum (laying crosses), as well as the fitted model (thick blue lines) and its 95% confidence intervals (blue error bars). Effect of **c** number of hunters on hunt success likelihood in wild chimpanzees, shown in blue are the observed probabilities to succeed in hunting (larger point areas denote a larger number of observations) as well as the fitted model (dashed lines). *P < 0.05, ***P < 0.001

Overall, 80% of all adult beggars received a share of meat (97% of hunters and 70% of non-hunters; ranging from 2 to 15 individuals present: 9.27 ± 3.5). In 84% of successful hunt cases, individuals trapped a single monkey, which for approximately half of the time was an adult individual (52%). Hunt participation had a significant effect on meat accessibility (access model full-null model comparison—likelihood ratio test: *χ*^2^ = 21.978, df = 1, *P* = 2.75 × 10^−6^; Fig. 1a, Table 1), and hunters (excluding catchers) were more likely to receive a share than bystanders. We found that older subjects (*P* = 0.003) gained more access to meat. Moreover, meat accessibility significantly increased with an increase in the size of the captured prey (i.e., young or adult; *P* = 0.003) and a decrease in overall fruit availability (*P* = 0.045). The dominance rank, sex, sub-group size, number of hunters, group identity, and the number of monkeys captured had no influence on meat distribution, although the latter was associated with some uncertainty (see Methods). The overall variance explained by the fixed effects was *R*^2^_m_ = 0.57.

**Table 1:** Effect of hunt participation on the likelihood to access meat

Term	Estimate	SE	CI_lower_	CI_upper_	*χ* ^2^	*P*
Intercept	2.619	0.742	1.281	26.281	—	—
Hunt participation [yes]	3.867 (47.815)	1.063	2.126	29.097	21.979	2.75 × 10^−6^
Age	1.299 (3.665)	0.447	0.502	11.235	8.997	0.003
Fruit availability	−0.987 (0.372)	0.513	−8.217	0.060	4.022	0.045
Prey size [young]	−2.765 (0.062)	0.924	−26.666	−0.938	9.030	0.003
Prey number [two]	1.652 (5.218)	1.844	−1.289	18.380	1.086	0.297
Number of hunters	0.750 (2.116)	0.515	−0.276	7.224	2.320	0.128
Sub-group size	−0.215 (0.806)	0.484	−2.188	1.157	0.258	0.612
Sex [male]	0.873 (2.393)	0.734	−0.861	8.497	2.659	0.103
Dominance rank	0.431 (1.539)	0.377	−0.594	4.520	1.292	0.256
Group [South]	0.097 (1.101)	1.056	−2.252	2.355	0.001	0.974

In parenthesis shown are the estimates as odds ratio. Categories of factors are indicated in brackets. Continuous variables are *z*-transformed, mean ± SD of the original variables: age 21.62 ± 10.03, fruit availability 1.82 ± 1.05, number of hunters 3.77 ± 1.39, sub-group size 9.27 ± 3.55, and dominance rank 0.59 ± 0.26 (range 0–1, with 1 being the highest social rank in each sex category)

Nonetheless, if hunt participation is confounded with motivation in acquiring meat, hunters may simply be more persistent beggars and acquire meat through harassment^42^. Hunters and bystanders equally harassed meat possessors in 18% of begging bouts, and begging duration (s) was 77.05 ± 102.15 and 120.61 ± 149.14 for hunters and bystanders, respectively. We found that motivation to acquire meat was not higher in hunt participants, as neither the duration of begging (Estimate ± SE: 0.073 ± 0.253; *P* = 0.772; bystanders as the reference category) nor the occurrence of harassment behavior (Estimate ± SE: −0.879 ± 0.607; *P* = 0.173) differed between hunters and bystanders.

### Hunt patrols and hunting success

Chimpanzees engaged in hunt patrols 72 times prior to initiating a hunt (54%), on 55 different days, with the longest hunt patrol duration from initiation until the first hunt attempt measuring approximately 4.75 h (mean ± SD: 1.18 ± 1.28 h). The sub-group size during hunting events were 5.88 ± 2.87 with and 5.40 ± 2.83 without prior hunt patrol behavior, and the total number of hunters was 3.45 ± 1.53 with and 2.6 ± 1.3 without hunt patrols. On 60% of days with hunt patrols, the first hunt attempt was successful, and in 19 out of the 22 (86%) days with a failed hunt first, individuals persisted with the search for a different monkey group and re-initiated hunting. This is in comparison to re-initiating hunting following a failed hunt on 10% of days when there was no prior hunt patrol. Overall, daily success rates were at approximately 85% for days with hunt patrols, in comparison to 47% success on days without hunt patrols (Table 2).

**Table 2:** Hunting events and daily success rates with and without hunt patrol behavior

	Events			Days		
	All	Successful	Unsuccessful	All	Successful	Unsuccessful
Total	143	80	63	118	78	40
With hunt patrols	72	47	25	55	47	8
Without hunt patrol	60	25	35	53	25	28
Unknown if patrolled	11	10	1	10	9	1

When testing whether hunt patrols and number of hunters influenced the likelihood to capture prey, the full-null model comparison was significant (success model—likelihood ratio test: *χ*^2^ = 22.939, df = 2, *P* = 1.04 × 10^−5^; Table 3). Both engaging in a hunt patrol prior to hunt initiation (Estimate ± SE: 1.088 ± 0.477; *P* = 0.020; Fig. 1b) and the number of hunters (Estimate ± SE: 0.923 ± 0.284; *P* = 0.0004; Fig. 1c) had a positive significant effect on hunt success likelihood. Moreover, we found a significant effect (*P* = 0.047) of South group being more successful in hunting than the East group. The sub-group size during the hunt, the presence of fully tumescent females, forest wetness, and general fruit availability had no influence on hunt success likelihood. The overall variance explained by the fixed effects was *R*^2^_m_ = 0.32.

**Table 3:** Effect of hunt patrols and number of hunters on hunt success

Term	Estimate	SE	CI_lower_	CI_upper_	***χ*** ^**2**^	*P*
Intercept	−1.127	0.485	−6.308	−0.285	—	—
Number of hunters	0.923 (2.516)	0.284	0.466	7.191	12.369	0.0004
Hunt patrol [yes]	1.088 (2.969)	0.477	0.175	5.290	5.437	0.020
Group [South]	1.058 (2.881)	0.547	−0.016	3.323	3.938	0.047
Sub-group size	−0.009 (0.990)	0.281	−0.644	0.594	0.001	0.973
Forest canopy wet [yes]	0.422 (1.525)	0.650	−1.056	2.368	0.425	0.515
Sexual swelling status [full tumescence]	0.294 (1.341)	0.554	−0.919	2.116	0.283	0.595
Fruit availability	0.252 (1.286)	0.256	−0.277	1.197	0.985	0.321

In parenthesis shown are the estimates as odds ratio. Categories of factors are indicated in brackets. Continuous variables are *z*-transformed, mean ± SD of the original variables: number of hunters 3.11 ± 1.5, sub-group size 5.71 ± 2.85, and fruit availability 1.85 ± 1.12

### Hunt patrols and urinary oxytocin levels

When investigating whether the behavior observed during hunt patrols was associated with oxytocinergic system reactivity, we found that urinary oxytocin levels were positively influenced by the target behaviors sampled (oxytocin model full-null model comparison—likelihood ratio test: *χ*^2^ = 21.168, df = 2, *P* = 2.53 × 10^−5^; Fig. 1d, Table 4). Specifically, hunting and hunt patrol behavior had a similar positive effect on oxytocinergic system activity but did not differ from each other (post hoc analysis: *z*-value = 1.626; *P* = 0.104). During both behaviors, individuals had significantly higher urinary oxytocin levels than during the control context (*P* < 0.001). Sub-group size and the dominance rank had no effect on urinary oxytocin levels; however, model results revealed a strong trend (*P* = 0.055) toward females having higher urinary oxytocin levels than males. Moreover, the second data collection period also revealed a positive significant effect (*P* = 5.61 × 10^−18^). The overall variance explained by the fixed effects was *R*^2^_m_ = 0.66.

**Table 4:** Effect of hunt patrols and hunts on urinary oxytocin levels in comparison to control context

Term	Estimate	SE	**CI**_**lower**_	**CI**_**upper**_	***χ*** ^**2**^	*P*
Intercept	1.728	0.257	1.202	2.213	—	—
Event [hunt]	0.805	0.161	0.473	1.136	21.169	2.53 × 10^−5^
Event [hunt patrol]	1.223	0.231	0.788	1.678		
Sex [male]	−0.269	0.139	−0.531	0.016	3.694	0.055
Dominance rank	−0.085	0.064	−0.223	0.044	1.706	0.192
Sub-group size	0.052	0.058	−0.062	0.170	0.268	0.605
Data collection period [second]	1.49	0.134	1.235	1.762	74.65	5.61 × 10^−18^

Categories of factors are indicated in brackets. Continuous variables are *z*-transformed, mean ± SD of the original variables: dominance rank 0.57 ± 0.28 (range 0–1, with 1 being the highest social rank in each sex category), sub-group size 9.2 ± 5.02

## Discussion

Chimpanzee behavior prior to and during hunting represents a useful model for investigating the evolution of human cooperative hunting. The main objectives of this study were to determine the extent of the cooperative nature of hunting in wild chimpanzees in Taï and the potential mechanisms facilitating future participation in cooperative acquisition tasks.

Theoretical models predict that cooperative hunting could evolve if hunters increase personal gain by hunting in groups in comparison to solitary hunts and if meat distribution benefits hunters over cheaters^17,26^. While in several social carnivore species per capita meat intake increases with the number of hunters^14^, the reward of labor is not well studied in non-human animals. Here we found very low success rates for single hunters. More importantly, we found that chimpanzee hunting success increased with the number of hunters, and that hunt participation was the strongest predictor of meat sharing across dominance ranks, ages, and sexes, in two different chimpanzee groups, supporting the cooperative acquisition hypothesis. These findings support previous work conducted on a different group (North group) in the same chimpanzee population >20 years earlier^17^, emphasizing that at least for the Taï chimpanzees reward of labor is a regular phenomenon across time and social groups. Importantly, our finding that chimpanzee labor during hunting is rewarded is unlikely to be an artifact of hunters being more motivated in acquiring meat or reaching the meat before bystanders, as hunters are often spread across ~50 m, often in different trees, while bystanders typically follow the hunt from the ground and are thus often quicker in reaching prey once captured. In fact, in all hunts with bystanders present and with more than two hunters, there was at least one bystander who was able to reach the meat possessor before all hunters did. Moreover, when comparing begging effort of hunters and bystanders we did not find a difference in begging duration or intensity between hunt participants and bystanders. It has been shown that variation in forest structure may influence hunting behavior^19,20^. For instance, the coordination of activities during a hunt may be especially important when in habitats with continuous tall canopy and thus numerous escape routes, such as the Taï forest^17,19,29^, emphasizing the importance of cooperation in hunting in this population. Although we did not investigate whether individuals coordinate hunt movements here, our results, that participation increases hunting success and is rewarded, suggest hunting in Taï is a cooperative act.

Despite hunt participation being the best predictor explaining meat distribution, we found that the majority of adult bystanders (70%) received meat, pointing out that the meat sharing network is widespread, and includes many non-hunters gaining access to the benefits provided by successful hunting (cheaters). Overall, the likelihood to receive meat increased with an increase in the age of subjects and larger prey and a decrease in general fruit availability. In terms of age, older male chimpanzees have been suggested to be the more skilled hunters^22,29^. Similarly, hunting performance in females might not only increase with age but also older female chimpanzees may be considered more attractive mates^43^, and potential mates receive more meat in some^44^ but not all chimpanzee populations^45^. Whether older males and females are more likely to access meat due to enhanced hunting or begging skills, other social factors or all is not tested here. If considering repeated interactions, reward for labor may extend beyond a specific hunting event. For instance, bystanders who are considered better hunters (older individuals) may gain a share as a means of facilitating future participation, a possibility which remains to be tested.

Meat sharing increased when larger prey was captured, likely due to the larger quantities of meat available for sharing, as suggested for large-sized game items in humans^11^. Sharing did not increase with an increase in prey items, despite having more catchers and hence initially more meat possessors present. Moreover, although we did not find an effect of fruit availability on hunt success, fruit availability affected overall meat distribution, and more individuals received a share of the meat with decreasing levels of general fruit availability. Whether lower general fruit availability leads to increased meat accessibility due to group members being more motivated in obtaining meat and/or due to changes in sharing inclinations of possessors remains to be tested.

When investigating hunting success, we found that chimpanzee participation in hunt patrols increased hunt success. Although hunt patrol is a behavior observed in different chimpanzee long-term field sites and occurring in more than half of the cases in our study, it is not well studied. During hunt patrol days compared with days without hunt patrols, failed hunt attempts were more often followed by further hunts, and daily success rates doubled during days in which individuals engaged in this coordinated group activity. These results indicate that motivation for hunting is higher on days in which chimpanzees jointly search for monkeys. Although we cannot address causality here, whether high motivation precipitates hunt patrols, hunt patrols build motivation, or both, experimental and observational studies in humans^32,33^ suggest that prior coordinated behavior improves motivation and induces a collective spirit in tasks that require dynamic responses to others in order to achieve a goal, for instance, synchronous marching before combat^32^. Moreover, temporal synchronization of actions in humans is suggested to improve not only motivation but also to promote skills and performance in joint-action tasks^32^. Although hunt patrols do not involve temporal synchronization of actions, we found that they increased hunt success independently of the number of hunters, raising the question as to whether hunt patrols increase hunting performance.

We found increased oxytocinergic system activity in chimpanzees while engaged in group searches for monkeys and hunting behavior in comparison with controls involving no social behavior. In a previous study, urinary oxytocin levels after hunting were higher in comparison with both non-social and social (i.e., multi-partner grooming) contexts, and multi-partner grooming per se was not associated with oxytocinergic system activity^41^. Here urinary oxytocin levels during hunt patrols were not different to hunting behavior, suggesting a link between the coordinated act and oxytocinergic activity. These results support recent evidence showing oxytocinergic involvement in other coordinated acts in chimpanzees such as territorial border patrols and intergroup encounters^41^. If coordinated behavior facilitates solidarity, motivation, and potentially performance leading to increased success, then a potential proximate mechanism such as the oxytocinergic system, operating in hunt patrols, would likely lead to reproductive benefits and be under positive selection.

Hunt patrol behavior is considered an indicator of an upcoming hunt, at times initiated hours prior to hunt attempts^27,34^, and is therefore suggested to involve planning^27,34^. Future planning is the ability to anticipate future needs separately from current ones^46^. It is a cognitively demanding process given the delay between action performance and access to reward^47^. Whether non-human animals are capable of future planning is debated^46,47^, and one may argue that individuals on hunt patrols merely satisfy immediate hunger needs, despite the delay between action and reward. However, as chimpanzees on hunt patrols pass numerous feeding trees but do not engage in feeding behavior, and given that hunt patrols are thought to more frequently occur in times of high food availability^19^, this seems unlikely. Moreover, we found that chimpanzees on hunt patrols are persistent in the sense that in most cases (86%) individuals re-initiate secondary hunts if the first one failed, as opposed to 10% on days without hunt patrols, suggesting that a common, planned goal is to catch a monkey. Given the latency between hunt patrol and hunt initiation, persistence in hunting, and higher chance in accessing benefits if performed, hunt patrols are a potential behavior for examining future planning in non-human animals.

Hunting and the sharing of meat are central in debates regarding the evolution of humans’ life history traits and social structure, with reward of labor suggested to facilitate future hunt participation to enhance hunting success as well as the production of extensive networks of commodity exchange^1–4^, hence making the availability of nutritionally valuable meat more reliable. Accordingly, group hunting and meat sharing are suggested to be fundamental in supporting the energetically demanding human life history adaptations, such as prolonged juvenile dependence, short inter-birth intervals, and increased survival^4^.

In the application of a comparative approach, we find evidence of hunt participation leading to higher hunting success and that labor in hunting is rewarded with meat returns in two groups of Taï chimpanzees. This indicates that hunting is a cooperative act. In terms of potential mechanisms supporting motivation and performance in group hunting and meat distribution, we find that both hunt success and the subsequent distribution of meat are likely enhanced by prior group coordinated activity during hunt searches, a prevalent component in reinforcing human collective group activity. Furthermore, we suggest that, in chimpanzees, the oxytocinergic system, a highly conserved mechanism involving an intrinsic emotional response, may facilitate joint participation in hunting and access to hunting rewards. Although not yet tested to our knowledge, it seems likely that this neurobiological mechanism should similarly facilitate cooperative hunting in humans.

Our results indicate that group-level cooperation, rare in the animal kingdom, was likely already embedded in the behavior and neurophysiology of one of our last common ancestors with the chimpanzee. This raises the question of whether selection pressures acting on group cooperative behavior have already shaped certain life history traits in our last common ancestor and perhaps may be in operation in one of our closest living relatives, chimpanzees. Thus chimpanzees serve as an important model species for investigating not only the evolution of dyadic or group cooperative acts but also the implications of group-level cooperation in shaping life history traits and enhancing survival, which is considered as uniquely human.

## Methods

### Data collection

Fieldwork was conducted at the Taï National Park, Côte d’Ivoire (5°45′N, 7°7′W) over two field seasons between October 2013–May 2014 and September 2014–May 2015 on two well-habituated chimpanzee groups (i.e., East and South). During the study period, the adult group size varied between 17 and 21 adult individuals in the South group and between 15 and 18 adult individuals in the East group, as a result of several deaths and immigration events. We collected all day focal animal sampling^48^ data of all adult males and a subset of the most habituated parous females (5 males and 5 females in each group, between 12 and 49 years of age), using the CyberTracker software (v3.389). Chimpanzees live in a fission–fusion social system in which individuals from the same group split into smaller and dynamic sub-groups of varying size and composition;^25^ we therefore continuously recorded data on sub-group composition and size. Furthermore, we determined the dominance relationships within each group using uni-directional submissive pant grunt vocalizations and estimated the dominance hierarchy both across the group and for both sexes separately (as dominance rank and sex are confounded variables in chimpanzee societies, with females subordinate to adult males), standardized to a range from 0 to 1, by applying a likelihood-based adaptation of the Elo rating approach^49^. Overall, we recorded a total of 2278 observation hours in the East group and 2271 in the South group during 557 focal days.

During focal follows, we documented every occurrence of hunt patrols and hunting behavior observed. Upon hunt initiation, all available observers (researchers and trained field assistants) interrupted focal follows and switched to the collection of all occurrence data on hunt participation and meat sharing of all adult individuals present in the sub-group. Moreover, to aid the collection of all occurrence data during hunting and meat sharing we used a Dictaphone and a HD Panasonic camcorder. Incorporating video data, we documented hunting and meat sharing behavior of 35 adult individuals (10 males and 25 females). The chimpanzee-to-human observer ratio (mean ± SD) was 2.84 ± 1.70 during hunting event and 3.41 ± 2.39 during meat sharing events.

All methods were non-invasive and were approved by the Ministries of Research and Environment of Côte d’Ivoire, and Office Ivoirien des Parcs et Réserves. All aspects of the study comply with the ethics policy of both the Max Planck Society and the Department of Primatology of the Max Planck Institute for Evolutionary Anthropology, Germany (www.eva.mpg.de/primat/ethical-guidelines.html) and the American Society of Primatologists principles for the ethical treatment of non-human primate.

### Hunt patrols and hunting

To evaluate the degree of opportunism involved in hunting, we recorded the occurrence of hunt patrols, a behavior described as a potential indicator of an incipient hunt^27,34^. During hunt patrols, individuals display a distinctive suite of behaviors. Chimpanzees on hunt patrols travel cohesively, slowly, with frequent pauses, and rarely forage or vocalize, similar to behaviors observed during territorial border patrols^34^. Unlike border patrols, hunt patrols often take place within the group’s territory, away from border areas, and rather than sniffing the ground for indirect cues of neighbor presence or listening at key vantage points in the periphery, individuals scan the forest canopy while alert to sounds or movements of potential monkey prey and change their travel direction accordingly^27,34^.

The start of a hunt patrol was recorded whenever the sub-group exhibited the suite of behaviors described. As opposed to other long-term field sites where chimpanzees successfully hunt and consume different mammalian orders such as other primates, ungulates and rodents^19,21,25,50^, in the Taï Forest the chimpanzees nearly exclusively hunt monkey species^25,34^. Therefore, hunts in Taï take place in the forest canopy that requires hunters to ascend trees in order to participate in hunting. The start of a hunt was therefore recorded whenever at least one individual climbed and moved toward the monkeys while in the forest canopy. The end of a hunt was recorded when all hunters had returned to the ground, no longer engaging in any hunting behavior with the same monkey group, or when a monkey was captured. We distinguished between hunters, bystanders, and catchers, while taking into account changes in activity during a hunt^34^, defined as: (i) hunters—any individuals playing an active role in approaching or chasing prey at canopy height where the prey were present, at any point during the hunt; (ii) bystanders—any individuals present in the sub-group during the hunt who throughout the entire hunt did not participate in climbing or chasing monkeys, nor captured prey; (iii) catchers (i.e., captors)—any individuals capturing prey either in the canopy or on the ground (as happens when monkeys slip during the chase or are knocked or pulled out of the tree by a chimpanzee in the canopy). Although hunting roles have been formerly described in Taï^29^, we neither collected data on specific hunting roles (e.g., ambusher, chaser, blocker) nor addressed it in this paper.

It has been formerly argued that, owing to varying forest visibility, large size of sub-groups and number of hunters, and the dynamic nature of chimpanzee hunting, it is difficult to track the activity of all individuals and discern bystanders from hunters^19^. Accordingly, several studies have used the number of males present in the sub-group as an estimate for the number of hunters^19,23,24^, despite likely overestimating the number of hunters^19^. The relative small group sizes and number of males (ranging between 15 and 21 adult individuals, with 5 adult males in each group) and the primary rainforest in Taï, together with the number of observers present (on average 1 observer per 3 adult chimpanzees during hunting events), allowed us to reliably distinguish hunters from bystanders in the majority of hunts. Whenever field conditions permit, distinguishing hunters from bystanders is far more accurate estimation for the number of hunters than using the number of males present, especially since females as well participate in hunting^25^.

Altogether, during hunt patrols and hunts we documented the identity of all participants, their role during the hunt (i.e., catchers, hunters, and bystanders), whether hunting behavior led to successful capture of prey, and if so, marked the number and age class of captured prey. We noted the identity of every individual begging and receiving access to meat and the way they attained the meat (i.e., sharing or scrounging). Sharing was documented whenever an individual obtained meat that was in possession of another, usually following begging behavior, and scrounging was documented whenever an individual collected a piece of meat or bone from the ground. Beggars were defined by their proximity to the meat possessor following Gilby et al.^42^. However, owing to small group sizes in Taï, we employed a more conservative approach and instead of 3 m^42^ we used 1 m proximity. Nonetheless, in our dataset these two methods provided identical results for the identity of beggars. To assure that we could reliably discern hunters from bystanders, we only analyzed hunting events for which we had all information regarding the activity of all adult individuals present. Overall, a total of 143 hunts were documented at a rate of 1 every 3 days in East and 1 every 8 days in South.

### Sharing under pressure and begging effort

The sharing under pressure hypothesis^10,42,51^ propose that possessors share food to decrease energy expenditure-related costs created by beggars and that the most persistent beggars are the most successful in obtaining a share. If participation in hunting is strongly dependent on individuals’ motivation in acquiring meat, then difference in meat accessibility between hunters and bystanders may be an artifact of begging motivation. We used video data of 24 meat-sharing events (starting when a possessor first acquired meat until the end of feeding time) with comprehensive video recordings of all begging and sharing behavior. We coded 149 dyadic begging bouts and extracted information regarding begging duration and intensity for every beggar present. We noted the onset of a begging bout when a beggar was in at least 1 m proximity and facing the meat possessor and ended when begging behavior ceased, either due to accessing meat or departure of either the beggar or possessor. As we are interested in factors affecting meat accessibility (y/n), we coded all consecutive begging interactions as separated dyadic begging bouts, until either sharing occurred or begging ended. During each begging bout, we recorded the duration of begging and whether harassment occurred. Harassment takes place when the costs of defending a resource are higher than those of sharing it, either due to reduced feeding efficiency or increased energy expenditure^42^. In Taï, unlike that suggested in other sites^42^, not all contact gestures interfere with the possessors’ feeding behavior (for instance, when touching the tail of the monkey without pulling; Supplementary movie 1). Also, other non-contact begging gestures affect the possessor’s feeding behavior by causing the possessor to change feeding location (Supplementary movie 2). Thus we coded as harassment whenever a begging interaction altered the possessor’s feeding behavior or caused the possessor to change posture. Physical contact between the beggar and the carcass and/or possessor were not a sufficient nor a required condition for harassment in our data.

### Fruit availability index

We calculated a fruit availability index on a monthly basis separately for the East and South group home ranges following a previously established index for Taï chimpanzees^52,53^. We used data on the phenology of fruiting trees, tree density, and tree basal area of the 74 most frequently eaten species for the chimpanzees to calculate an index for each chimpanzee group on a monthly basis. Accordingly, the fruit availability index FA_*m*_, for month *m* is defined as follows:

$$\mathrm{FA}_{\it m} = \mathop {\sum }\limits_{{\it k} = 1}^{\it n} {\it D}_{\it k}B_{\it k}P_{{\it km}}$$

where *D*_*k*_ = the density of species *k* across the study area,

*B*_*k*_ = the mean basal area (measured by trunk diameter at breast height) of species *k* across the study area, and

*P*_*km*_ = the percentage of observed fruiting tree species *k* with mature fruits across the study area in month *m*.

### Urine sample collection and analysis

Focal follows were accompanied by the systematic collection of urine samples from the focal individual for oxytocin hormone analysis from leaf litter using a plastic pipette. We applied a targeted event sampling method in which urine samples were collected within 15–60 min after the target behaviors of group hunting and hunt patrols, according to the estimates of the oxytocin clearance rate^54,55^. We used a conservative time period of 90 min without any social interactions or cooperative behaviors as control samples. To reliably attribute hormone levels to the specific events, we did not analyze samples that included any social or cooperative behavior other than hunts and hunt patrols, for instance, due to overlap with meat sharing or grooming.

We performed a solid phase extraction with Chromabond^®^ HR-X SPE cartridges (1 mL, 30 mg) and analyzed samples using a commercial enzyme immunoassay kit (Assay Designs, Catalog No. 901-153A-0001). For the extraction, we first conditioned the cartridges with 1 mL 100% methanol followed by 1 mL distilled HPLC water. Thawed urine samples were vortexed (10 s) and centrifuged (1 min at 1000 rpm), then diluted 1:2 using 0.1% trifluoroacetic acid and loaded onto the cartridge. We then washed the cartridge with 1 ml of 10% acetonitrile containing 1% trifluoroacetic acid in water and eluted using 1 mL 80% acetonitrile. We evaporated sample extracts using air stream at 50 °C, reconstituted with 300 µL 100% ethanol, and vortexed for 10 s. Samples were left at 4 °C for 60 min and evaporated again using the same procedure. Following this, we reconstituted the dried sample in 250 µL assay buffer supplied in the immunoassay kit, vortexed (10 s), and centrifuged (1 min at 10,000 rpm). Samples were added to the assay as 100 µL duplicates following instructions of the assay provider.

We also measured creatinine levels in all urine samples and expressed urinary oxytocin values as pg/mg creatinine, to control for variation in urine volume and concentration^56^. Urinary oxytocin values were expressed as pg/mg creatinine, and all urine samples with creatinine levels ≤0.05 mg/mL were excluded (*n* = 3, <3% of the samples). The oxytocin assay standard curve ranged from 15.62 to 1000 pg/mL, assay sensitivity was 15 pg/mL, and all analyzed samples produced results within the linear range of the assay’s standard curve. We excluded four control samples that produced results under the linear range but for which there was no remaining material for re-measurement.

In a previous study, assay validations of parallelism and accuracy for chimpanzee urine were conducted and were satisfactory^55^. Inter-assay coefficients of variation of low (50 pg/mL) and high (250 pg/mL) value quality controls were 21.4% and 7.8% (*n* = 28 batches), respectively, while intra-assay coefficients of variation of low (50 pg/mL) and high (250 pg/mL) value quality controls were 14.2% and 9%, respectively. Overall, 106 urine samples from 10 subjects of the East group (10.6 ± 7.3 sample/subject) were included in the statistical analysis.

### Statistical analysis

To investigate which factors affect hunt success, meat accessibility, and motivation to acquire meat among adult chimpanzees, we conducted three Generalized Linear Mixed Models (GLMM) with Binomial error structure and logit link function and a Linear Mixed Model (LMM)^57^ with Gaussian error structure and identity link function.

To test the cooperative acquisition hypothesis, we examined how the likelihood of adult chimpanzees to access meat via sharing varied across hunters and bystanders by fitting a Binomial GLMM (access model)^57^, with meat access (no/yes) as the response and hunt participation (bystander/hunter) as the sole test predictor. We did not include the individuals that captured the prey (catcher) in the analysis to avoid inflating the number of hunters receiving a share. Moreover, to investigate whether hunters are more successful beggars than bystanders we did not include cases of scrounging or any adult individuals that were present during the meat sharing but did not beg. To investigate the influence of different variables on the likelihood of receiving meat, we included sex, age, and dominance rank of the subject; group identity (East and South); sub-group size; and the number of hunters. Moreover, in order to account for differences in the number of monkeys caught, prey amount, or variation in general fruit availability that might influence meat distribution, we controlled for prey number and size (i.e., young or adult monkey) and fruit availability using phenology data. When two or more monkeys were captured in a single hunt, the prey size was determined additively: if the cumulative size of prey was smaller than an adult (i.e., two infants), it was coded as young, or equal or larger than an adult (i.e., two juveniles or an adult and infant), it was coded as adult. We included subject and event identities as random effects to account for certain subjects or events disproportionally affecting meat accessibility results. Furthermore, to keep type I error rate at the nominal 5%, we included random slopes^58,59^ for dominance rank and age within event identity, and dominance rank, number of hunters, and fruit availability within subject. Our dataset for the sharing model included 303 data points of 32 individuals, of 53 different events.

To investigate whether hunters and bystanders differ in their motivation to acquire meat, we ran two sets of identical models with the response being either (a) begging duration (a Gaussian LMM; begging duration model) or (b) harassment occurrence (a Binomial GLMM; harassment model), with hunt participation as the sole test predictor. In the models, we controlled for sex, age, and dominance rank of the subject; group identity (East and South); and prey size. We accounted for subject and event identities as random effects and included random slopes for dominance rank and age within event identity. Our dataset for both models included 149 begging bouts of 24 meat eating events involving 26 individuals.

To investigate the hypothesis that the coordinated movements observed during hunt patrols and number of hunters enhance hunt success, we examined key ecological and social variables thought to influence the response, hunt success (yes/no). For 114 out of the 143 observed hunt attempts, we were able to document the identity of all hunters and bystanders as well as whether individuals engaged in a hunt patrol in the hour prior to hunt initiation. We fitted a Binomial GLMM (success model) with our test predictors as the number of hunters and whether the hunt followed a hunt patrol. We evaluated Variance Inflation Factors (VIF)^60^ to identify potential violations of model assumptions due to high collinearity for all predictors. Although occurrence of hunt patrols may be correlated with the number of hunters, VIF values (number of hunters: 1.433; hunt patrol: 1.173) did not reveal any issues, meaning that we were able to reliably test both predictors. We controlled for sub-group size^20,61^, group identity (East and South), and presence of fully tumescent females in the sub-group^20^. We as well controlled for ecological factors such as whether the forest canopy was wet (potentially increasing the likelihood for monkeys to slip and fall during the hunt)^25^ and fruit availability that has been shown to have an effect on the occurrence of hunting and hunt patrols^19,61,62^. We included the group nested in date as a random effect to account for specific days disproportionally affecting hunt success likelihood over others in the two different groups. We did not include any random slopes due to low variation within the random effect of date. Our dataset for the success model included 114 hunt data points from 93 different days.

Furthermore, we investigated whether the cohesive and coordinated behavior observed during hunt patrols is associated with oxytocinergic system reactivity by fitting a LMM (oxytocin model)^57^ with Gaussian error structure and identity link function. We measured urinary oxytocin levels (pg/mg creatinine) collected after (i) participation in group hunting of monkeys^17^. To prevent any influence of subsequent meat sharing on urinary oxytocin levels, we included samples collected before the individual received a share of meat or after unsuccessful hunts (Hunt: 3 males and 2 females, *n* = 18 samples); (ii) participation in hunt patrols (Hunt patrol: 5 males and 1 female, *n* = 19 samples); and control samples collected after (iii) feeding, resting, and traveling without positive social interactions, except for vocalizations (Control: 5 males and 5 females, *n* = 68 samples). In this model, we controlled for sub-group size, sex, and dominance rank by including them as additional fixed effects, to account for variation in urinary oxytocin levels due to these variables. Moreover, we included the data collection period as a fixed effect since the East chimpanzee group experienced social changes of unusual high rates of intergroup interactions in the second field season period, thought to influence urinary oxytocin levels. This model included only samples of East group chimpanzees due to low hunt patrol sample size (*n* = 2) for the South group, thus we did not control for group identity (although both groups participated in hunt patrols in similar frequencies). To account for certain subject or event identities having a disproportionate influence on the response, we included them as random effects. We also included random slopes^58,59^ for event type (after manually dummy coding and then centering), sub-group size, and rank within subject. We conducted a post hoc analysis to test the effect of each target behavior in relation to each other, using the function glht of the R package multcomp^63^. Our dataset for the oxytocin model included 106 samples from 10 different individuals from 85 different events.

We fitted all models in R (version 3.4.1^64^) using the functions lmer or glmer of the R package lme4^65^ and compared the fit of all full models with those of a respective null model lacking only the test predictors but otherwise being identical to the respective full model in all other terms^66^ using a likelihood ratio test. Prior to fitting the models, we checked all predictors and the response for their distribution and, as a consequence, log transformed begging duration and urinary oxytocin levels to achieve a more symmetrical distribution. We then proceeded by *z*-transforming the covariates of sub-group size, number of hunters, ranks, ages, and fruit availability. Visual inspection of qq-plots and residuals plotted against fitted values did not reveal obvious deviations from the assumptions of normally distributed and homogeneous residuals. We tested the significance of the fixed effects by systematically dropping them from the model one at a time^58^ and comparing the full with the respective reduced model lacking the individual fixed effect, using the drop1 function in R^64^. Model stability was assessed for all models by excluding the random effects one at a time and comparing the estimates derived for these data with those derived for the full data set. This indicated uncertainty in the estimates for prey number influenced by a single event. We derived confidence intervals by means of parametric bootstraps (function bootMer of the package lme4). We calculated effect sizes (*R*^2^) and report the variance explained by the fixed effects (marginal—*R*^2^_m_)^67^, using the function r.squaredGLMM from the R package MuMIn^68^. We used the function vif of the R package car^69^ applied to a standard linear model lacking the random effects to derive VIF, which did not reveal collinearity problems (largest VIF: oxytocin model: 1.13; success model: 1.60; access model: 2.03; begging duration and harassment model: 2.02^60^).

## Electronic supplementary material

Supplementary movie 1

Supplementary movie 2

Description of Additional Supplementary Items

## Data Availability

The datasets analyzed during the current study are available from the corresponding author upon reasonable request.
