# Supplementary material for: Reward of labor coordination and hunting success in wild chimpanzees
Source: Commun Biol. 2018 Sep 10;1:138. doi: 10.1038/s42003-018-0142-3 (PMC6131550; doi:10.1038/s42003-018-0142-3)
Supplement: Supplementary file 3 — Description of Additional Supplementary Items [file 42003_2018_142_MOESM3_ESM.docx]

Description of additional supplementary items –

Reward of labor coordination and hunting success in wild chimpanzees

Liran Samuni, Anna Preis, Tobias Deschner, Catherine Crockford, Roman M. Wittig

**Supplementary movie 1** – chimpanzee begging behavior involving physical contact but not interfering with the possessors’ feeding behavior

**Supplementary movie 2** – chimpanzee non-contact begging behavior that interferes with the possessors’ feeding behavior
